# Comparison of the Effectiveness of the DNIPRO Gen 2 and SICH Tourniquets Versus the CAT Gen 7 and SOFTT-W Gen 4 Tourniquets

**DOI:** 10.3390/medicina62040627

**Published:** 2026-03-26

**Authors:** Jakub Zachaj, Katarzyna Moorthi, Łukasz Kręglicki, Kateryna Bielka, Hanna Formina, Liliia Kryveshko, Robert Gałązkowski, Marcin Podgórski, Patryk Rzońca

**Affiliations:** 1 Clinical Department of Cardiac Surgery and Pediatric Surgery, Children’s Clinical Hospital, 02-091 Warsaw, Poland; jakub.zachaj@uckdsk.pl; 2 Polish Medical Air Rescue, 01-934 Warsaw, Poland; kmoorthi@lpr.com.pl; 3 Department of Emergency Medical Services, Faculty of Health Sciences, Jagiellonian University Medical College, 31-126 Cracow, Poland; lukasz.kreglicki@uj.edu.pl; 4 Department of Surgery, Anesthesiology and Intensive Care of Postgraduate Education, Bogomolets National Medical University, 01601 Kyiv, Ukraine; ekateryna.belka@gmail.com (K.B.); goanna226@gmail.com (H.F.); lilia.kryveshko01@gmail.com (L.K.); 5 Department of Emergency Medical Services, Faculty of Health Sciences, Medical University of Warsaw, 02-091 Warsaw, Poland; robert.galazkowski@wum.edu.pl (R.G.); marcin.podgorski@wum.edu.pl (M.P.); 6 Department of Human Anatomy, Faculty of Health Sciences, Medical University of Warsaw, 02-091 Warsaw, Poland

**Keywords:** haemorrhage, tourniquets, effectiveness, medical devices, self-application, application time

## Abstract

*Background and Objectives*: Massive extremity haemorrhage remains the leading cause of preventable death on the battlefield and among trauma victims in civilian settings. Tourniquets are an effective, low-cost tool used to rapidly control bleeding. However, the availability of certified tourniquet models during a full-scale armed conflict can be significantly limited. This favours the emergence of locally manufactured devices. The aim of this study was to compare the effectiveness of the DNIPRO Gen 2 and SICH tourniquets with the CAT Gen 7 and SOFTT W Gen 4 tourniquets recommended by the Committee on Tactical Combat Casualty Care. *Materials and Methods*: The study included 51 Ukrainian medics experienced in prehospital care. Application speed was measured with a stopwatch, and occlusion success was confirmed by Doppler ultrasound. Pain was measured using the NRS, and participants were also able to provide subjective comments regarding the ergonomics and design of the tourniquets. *Results*: The four tourniquets tested demonstrated different occlusion success rates in arm and leg application. In upper extremity application, the SICH had the highest success rate (98.0%) and was associated with higher odds of successful application compared with the SOFTT-W Gen 4 (OR 25.14). In lower extremity application, the CAT Gen 7 had the highest rate of success (94.1%) and was 7.5 times more likely to achieve occlusion than the SOFTT-W Gen 4 (OR 7.54). The DNIPRO Gen 2 was rated most painful (Median 6), with significantly lower pain levels reported for the SICH (Median 4), the CAT Gen 7 (Median 5), and the SOFTT-W Gen 4 (Median 4). *Conclusions*: The DNIPRO Gen 2 and SICH tourniquets demonstrated high occlusion success rates, comparable to the CAT Gen 7 and superior to the SOFTT-W Gen 4. These findings suggest that Ukrainian-manufactured tourniquets may demonstrate comparable performance to CoTCCC-recommended tourniquets in a simulated prehospital setting.

## 1. Introduction

Advances in battlefield medicine over the past two decades have led to the systematisation of the concept of preventable deaths and the identification of key mechanisms responsible for fatal outcomes among soldiers on the battlefield. The experience of the United States Armed Forces and NATO countries during the Global War on Terror (GWOT) and Counter Insurgency Operations (COINs) made it possible to define the following three major and potentially reversible causes of death in combat: massive extremity haemorrhage, airway obstruction, and tension pneumothorax. As a result, prehospital intervention strategies focused on haemorrhage control within the first minutes after injury were developed and implemented, with tourniquets playing a central role in this process [1].

The ongoing Russia Ukraine conflict, which escalated in February 2022, differs substantially in scale and character from GWOT and COINs. At the same time, available data from earlier Ukrainian military engagements, including the Anti-Terrorist Operation (ATO) in Donbas, indicate that massive extremity haemorrhage was the predominant cause of preventable death among Ukrainian soldiers. Tourniquets were used on a large scale, with the demand for them increasing sharply under full-scale war conditions [2]. Clinical reports confirm significant and growing use of tourniquets among wounded Ukrainian service members, with thousands of documented applications since the start of the war [3].

Currently, only a limited number of commercial tourniquets are recommended by the Committee on Tactical Combat Casualty Care (CoTCCC). These include: the Combat Application Tourniquet (CAT) Gen 7 and Gen 6 (recommended until fully replaced by Gen 7), the Ratcheting Medical Tourniquet—Tactical (RMT T), the SAM Extremity Tourniquet (SAM XT), the SOF Tactical Tourniquet—Wide (SOFTT-W) Gen 4, the Tactical Mechanical Tourniquet (TMT), the TX2” Tourniquet (TX2), and the TX3” Tourniquet (TX3) [4]. Persistent equipment shortages and logistical constraints prompted Ukrainian manufacturers to develop their own designs. As a result, the Armed Forces of Ukraine have widely adopted the following two domestically produced windlass tourniquets: the DNIPRO Gen 2 and the SICH. They currently serve as parallel alternatives to the CoTCCC recommended models. However, there are no published comparative data evaluating their effectiveness in achieving arterial occlusion, speed of application, or pain during application in a simulated prehospital setting.

Given the limited availability of data and the rapidly growing clinical demand, it is necessary to conduct studies assessing the quality and performance of Ukrainian tourniquets relative to standardised and widely used reference models. The aim of this study was to compare the DNIPRO Gen 2 and SICH tourniquets with the CoTCCC-recommended CAT Gen 7 and SOFTT-W Gen 4 tourniquets in terms of occlusion success rate, speed of application, ease of use, and level of pain associated with tourniquet application in a simulated experimental setting involving trained prehospital medical personnel.

## 2. Materials and Methods

### 2.1. Study Design

The study was designed and conducted as a prospective crossover experimental study carried out in a simulated prehospital setting during a tactical medicine training course for Ukrainian medics. The aim of the study was to assess the occlusion success rates and user-related performance parameters of two Ukrainian tourniquets, the DNIPRO Gen 2 and the SICH, in comparison to the CoTCCC-recommended CAT Gen 7 and SOFTT-W Gen 4 tourniquets. The study protocol included the evaluation of application speed, rate of occlusion success, level of pain associated with tourniquet application, and ease of use. It was designed to assess the performance of the tourniquets during the earliest phase of casualty care, specifically self-aid in the Care Under Fire (CUF) phase or at the Point of Injury (POI).

### 2.2. Participants

Healthcare professionals with clinical experience in prehospital medicine were invited to participate. Inclusion criteria were informed consent, medical education (physicians or feldshers—healthcare professionals in Ukraine with training comparable to paramedics), and clinical experience defined as active work in prehospital emergency medical service teams.

### 2.3. Devices

**Figure 1:**
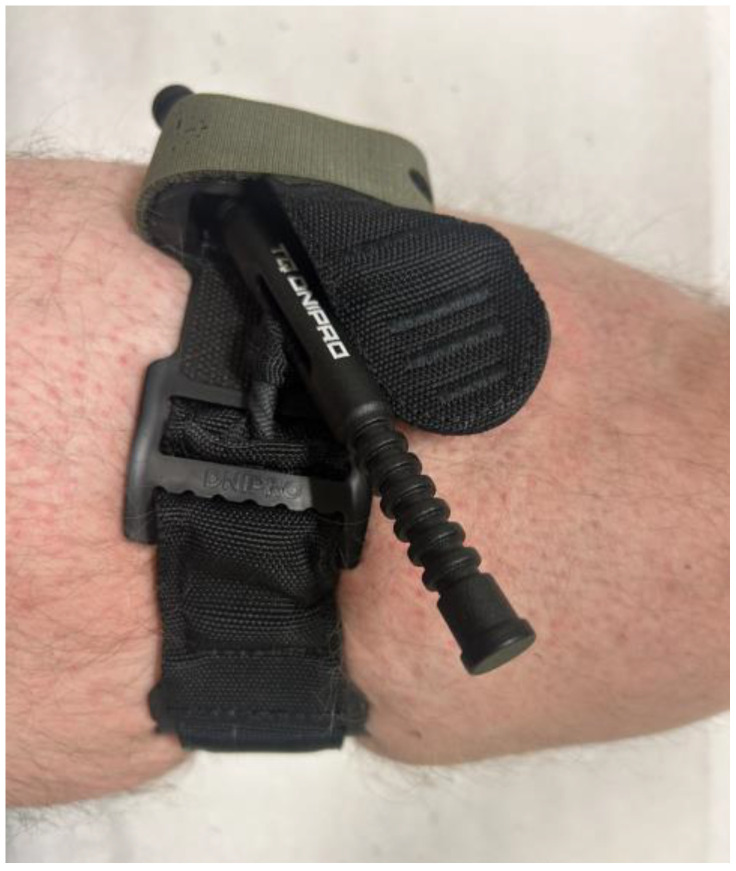
DNIPRODNIPRO Gen 2 tourniquet (TQ Dnipro, Kyiv, Ukraine).

**Figure 2:**
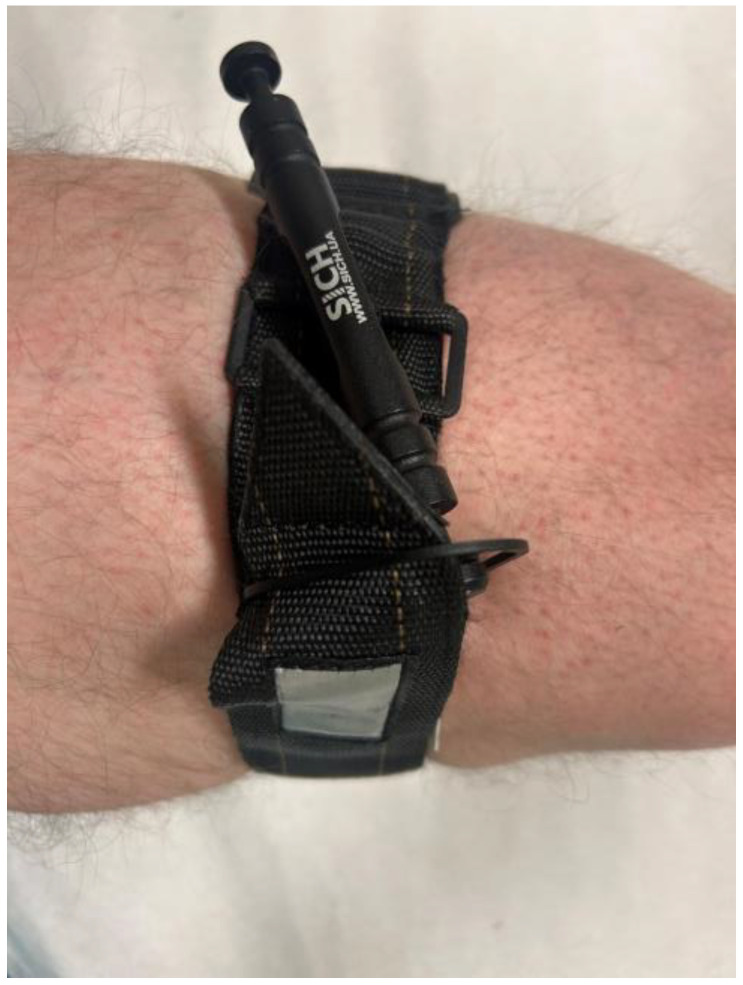
SICH tourniquet (Sich-Ukraine, Kyiv, Ukraine) applied at the mid-thigh.

The following four tourniquet models were used in the study:

• DNIPRO Gen 2 (TQ Dnipro, Kyiv, Ukraine) is a windlass tourniquet manufactured by TQ Dnipro. It consists of a synthetic strap covered with Velcro, allowing rapid circumference adjustment. The internal tensioning strap is tightened by rotating a windlass rod made of lightweight metal alloy, generating occlusion pressure. The circumference of the tourniquet is adjusted by threading the strap through a metal buckle with notches that prevent loosening after tension is applied. The windlass rod is attached to a plastic plate, and its textured surface facilitates manipulation, including when wearing tactical gloves. The locking mechanism consists of a U-shaped component that stabilises the rod once the strap is tightened, along with an additional Velcro strap that secures the windlass from above. The strap features perforated markings that are intended for recording the time of application (Figure 1) [5].
• SICH (Sich-Ukraine, Kyiv, Ukraine) is a windlass tourniquet developed by Sich Ukraine. It consists of a synthetic strap covered with Velcro, with a distinctive pattern of alternating loop-and-hook sections designed to ensure effective operation even when the Velcro surface becomes contaminated. The terminal portion of the strap is reinforced to facilitate rapid identification of the end and adjustment of the circumference of the tourniquet during application. The strap is threaded through a dual slot metal buckle, and occlusion pressure is generated by rotating a duralumin windlass rod that tightens the internal segment of the strap forming part of the tourniquet. The locking mechanism consists of a triangular plate with profiled grooves into which the windlass rod is inserted after tightening, then pressed toward the apex of the lock to stabilise it in position. The lateral holes in the locking element allow securing the loose end of the strap after application. The tourniquet has a coated tape sewn onto the strap end, on which the time of application can be indicated with a marker pen or any other sharp object (by scratching) (Figure 2) [6].
• CAT Gen 7 (C-A-T Resources LLC, Rock Hill, SC, USA) is a widely used CoTCCC-recommended windlass tourniquet employed in both military and civilian prehospital systems. It consists of a synthetic strap covered on one side with Velcro. The circumference of the tourniquet is adjusted by threading the strap through a single slot buckle, enabling rapid fitting to the limb. Occlusion pressure is generated by rotating the windlass rod, which tightens the internal segment of the strap forming part of the tourniquet. The rod has profiled grooves that facilitate manipulation, including when used with tactical gloves. The locking mechanism consists of a U-shaped plate that secures the rod in place, with additional stabilisation provided by a Velcro strap, which closes over the windlass from above and provides a space for recording the time of application. The terminal portion of the strap is marked in red to facilitate rapid identification and gripping of the end of the tourniquet [7].
• SOFTT-W Gen 4 (Tactical Medical Solutions, LLC, Anderson, SC, USA) is a windlass tourniquet that consists of a durable synthetic strap without Velcro, which distinguishes its design from that of the other tourniquets used in the study. The circumference of the tourniquet is adjusted by threading the end of the strap through a metal buckle attached to the windlass section. The necessary occlusion pressure is generated by rotating a metal windlass rod that tightens the strap as it passes through a specially formed structural element. The rod is stabilised by a movable U-shaped locking element, supported by an additional triangular securing element. The strap is reinforced with textile layers on the upper surface and a rubberised layer on the underside, improving stability and preventing slippage on the skin. The time of application can be recorded on a sewn-on tape located on the external portion of the device [8].

### 2.4. Procedures

Each participant completed a one-hour training session that included a demonstration of all the four tourniquets tested and self-application practice in accordance with CoTCCC guidelines (Care Under Fire/Point of Injury). Participants were instructed to apply the tourniquet in a “high and tight” position. For the upper extremity, the tourniquet was applied directly on bare skin, whereas for the lower extremity it was applied over clothing. Participants then applied the tourniquets under conditions simulating prehospital self-aid. Each participant applied four tourniquet models to one selected upper and one selected lower extremity, resulting in eight applications per participant (408 trials overall). All four tourniquet models were applied to each of these limbs in a fixed sequence (CAT Gen 7, DNIPRO Gen 2, SOFTT-W Gen 4, and SICH). Due to organisational constraints associated with conducting the study in Kyiv during a period of repeated air raid alerts, the study procedure was shortened compared with the initial protocol assumptions. Tourniquet application was considered complete when the participant secured the windlass in the locking mechanism. Application time was defined as the interval from initial placement of the device on the limb to this point. The decision to stop tightening the tourniquet was left to the participant’s judgement, in accordance with standard training principles. No mechanical failures of the tourniquets were observed during the study. After each trial, the devices were inspected for signs of damage or malfunction. Although the participants were healthcare professionals involved in prehospital care, most reported limited routine use of tourniquets in their daily clinical practice.

### 2.5. Outcomes

The primary endpoint of the study was successful arterial occlusion with tourniquet application, as confirmed by ultrasound examination using a portable Butterfly iQ+ ultrasound device (Butterfly Network Inc., Guilford, CT, USA) [9]. Arterial occlusion was assessed after completion of tourniquet application. Examinations were performed by experienced ultrasound operators who assessed arterial blood flow in distal arteries. For the upper extremity, blood flow was evaluated at the level of the radial artery at the wrist. For the lower extremity, blood flow was assessed at the dorsalis pedis or posterior tibial artery. Doppler assessment was initiated immediately after completion of tourniquet application and continued for up to one minute. The absence of a detectable Doppler signal during this period was considered confirmation of successful arterial occlusion. The examiner performing the Doppler ultrasound assessment was aware of the type of tourniquet applied, as the devices differed visually and blinding was not feasible during the procedure. Secondary endpoints included:

1. speed of application, measured in seconds;
2. pain during tourniquet application, assessed using the 11-point NRS scale (0–10) [10] (assessed immediately after tourniquet removal);
3. number of windlass turns required to achieve arterial occlusion;
4. ease of use, assessed using a 5-point Likert scale (1 = very difficult, 5 = very easy) (recorded immediately after tourniquet application).

Rest periods were provided between applications to prevent cumulative pain and fatigue. The intervals were not standardised and were adjusted individually for each participant, typically lasting several minutes until the discomfort subsided. No participant discontinued the study due to excessive pain. All participants were familiar with the NRS scale, and its scoring instructions were reviewed prior to measurements to enhance the reliability of the results.

### 2.6. Ethics

The study was conducted in accordance with the guidelines of the Declaration of Helsinki. The study protocol was approved by the Ethics Committee of the Bogomolets National Medical University (protocol #148, 7 September 2021). All participants provided informed consent after receiving information about the purpose and procedure of the study and the potential discomfort associated with the application of tourniquets. Participants were free to withdraw from the study at any stage without providing a reason.

### 2.7. Statistical Analysis

Statistical analysis was carried out using IBM SPSS Statistics v.29 (IBM Corp., Armonk, NY, USA). Categorical variables were reported as numbers (*n*) and percentages (%), whereas continuous variables were reported as medians (Mdn) and interquartile ranges (IQRs).

Because each participant performed multiple tourniquet applications, the observations were not independent but clustered within individuals. To account for this repeated-measure structure, mixed-effect models were used. For the binary endpoint of arterial occlusion confirmed by Doppler ultrasound, mixed-effect logistic regression models with a binomial distribution and logit link function were applied, with participant included as a random intercept. Separate models were fitted for upper and lower extremities, with the SOFTT-W Gen 4 tourniquet used as the reference category.

For continuous outcomes, including application time, number of windlass turns required to achieve occlusion, pain intensity (NRS score), and ease-of-use rating, linear mixed-effect models were applied with participant included as a random intercept and tourniquet type included as a fixed effect.

Effect estimates for binary outcomes are presented as odds ratios (ORs) with 95% confidence intervals (95% CIs). A two-sided *p* < 0.05 was considered statistically significant.

## 3. Results

A total of 51 Ukrainian healthcare professionals participated in the study, the majority of whom were male (72.5%) and physicians (68.6%).

The tourniquets differed in the rates at which they achieved arterial occlusion, as confirmed by Doppler ultrasound. In arm application, the SICH demonstrated the highest success rate (98.0%), followed by CAT Gen 7 (94.1%) and DNIPRO Gen 2 (94.0%), whereas SOFTT-W Gen 4 showed the lowest success rate (61.7%). In leg application, CAT Gen 7 achieved the highest success rate (94.1%), followed by SICH (86.3%) and DNIPRO Gen 2 (80.4%), while SOFTT-W Gen 4 again demonstrated the lowest success rate (68.0%).

Application time for the upper extremity varied between devices, with median values ranging from 32 s for CAT Gen 7 and SICH to 36 s for DNIPRO Gen 2. In the lower extremity, application time varied more markedly between devices, with CAT Gen 7 demonstrating the shortest median application time (29 s) and SOFTT-W Gen 4 requiring the longest time (38 s).

Pain intensity during tourniquet application was similar between devices for the upper extremity. However, a small difference was observed in the lower extremity, with DNIPRO Gen 2 associated with slightly higher median pain scores compared with the other tourniquets.

Differences were also observed in the number of windlass turns required to achieve arterial occlusion. CAT Gen 7 required the fewest turns in both arm (median two turns) and leg application (median three turns), whereas SICH and SOFTT-W Gen 4 required a higher number of turns.

Ease-of-use ratings varied between tourniquets for both arm and leg applications. CAT Gen 7 received the highest ratings, whereas SOFTT-W Gen 4 received the lowest scores. Detailed descriptive results are presented in Table 1.

Mixed-effect logistic regression analysis confirmed significant differences in the odds of achieving arterial occlusion between the tested tourniquets (Table 2). In upper extremity application, DNIPRO Gen 2 (OR = 9.66; 95% CI: 2.60–36.85), CAT Gen 7 (OR = 9.85; 95% CI: 2.66–36.55), and SICH (OR = 25.14; 95% CI: 3.71–170.21) were all significantly associated with successful arterial occlusion compared with the reference device SOFTT-W Gen 4. In lower extremity application, CAT Gen 7 (OR = 7.81; 95% CI: 2.08–39.39) and SICH (OR = 3.04; 95% CI: 1.11–8.34) remained significantly associated with successful arterial occlusion compared with SOFTT-W Gen 4, whereas DNIPRO Gen 2 did not differ significantly from the reference device.

Linear mixed-effect models further demonstrated differences between tourniquet models for several secondary outcomes (Table 3). SOFTT-W Gen 4 required significantly longer application time in the lower extremity compared with the other devices. No significant differences were observed between devices for application time in the upper extremity. Pain intensity did not differ significantly between tourniquets for arm application, whereas in the lower extremity DNIPRO Gen 2 was associated with higher pain scores compared with SOFTT-W Gen 4 and SICH. The number of windlass turns required to achieve arterial occlusion also differed significantly between devices, with SICH and SOFTT-W Gen 4 requiring more turns than CAT Gen 7 and DNIPRO Gen 2. Ease-of-use ratings differed significantly between devices, with CAT Gen 7 receiving the highest usability scores and SICH and SOFTT-W Gen 4 receiving the lowest ratings. Estimated marginal means and Bonferroni-adjusted pairwise comparisons are presented in Table 3.

## 4. Discussion

The effectiveness of CoTCCC-recommended tourniquets in controlling massive extremity haemorrhage is well-documented in both tactical and civilian settings, which is further supported by their widespread use during the ongoing Russia–Ukraine conflict [11,12,13]. Contemporary systematic reviews indicate improved survival following their prehospital application [14,15]. Logistical challenges, including limited access to CoTCCC-recommended tourniquets, have led to the development of domestic alternatives on the Ukrainian market. The DNIPRO Gen 2 and the SICH were designed as widely accessible tourniquets for the armed forces and emergency responders. They are both based on a windlass mechanism that allows the user to apply occlusion pressure by twisting the strap, which makes them functionally similar to the CAT Gen 7 and the SOFTT-W Gen 4.

The Combat Application Tourniquet is currently the most widely used and best studied tourniquet worldwide. Its development began in the late 1980s under a U.S. Department of Defence programme, with numerous design modifications resulting in a device that is easy to use, effective, and suitable for self-application [16]. In the present study, the CAT Gen 7, the DNIPRO Gen 2, and the SICH demonstrated similar occlusion success rates, with the SOFTT-W Gen 4 being significantly less effective in achieving occlusion. Notably, in lower extremity application the CAT Gen 7 demonstrated a higher occlusion success rate than the DNIPRO Gen 2, with a difference of approximately 14 percentage points. Although this difference should be interpreted cautiously given the simulated experimental design, it may have potential clinical relevance and warrants further investigation in larger and more realistic settings. This observation is consistent with findings from a study by Katsnelson et al. (2020), who demonstrated the superiority of the CAT7 over the SOFTT W in lower extremity haemorrhage control under laboratory conditions [17]. Similarly, in a study by Treager et al., the CAT achieved pulselessness more quickly and more consistently than the SOFTT W and the Tactical Mechanical Tourniquet [16]. Similar results were found by Potac et al., who reported a 95% application success rate for the CAT Gen 7 and a significantly lower rate for the SOFTT [18]. Our findings, obtained from 51 trained prehospital healthcare professionals, confirm these observations and extend them to include the DNIPRO Gen 2 and the SICH tourniquets, demonstrating that their effectiveness is similar to that of the CAT Gen 7 and clearly superior to the SOFTT-W Gen 4. The tourniquets tested share several design features, including a similar strap width and the windlass tightening mechanism. These characteristics allow the devices to generate sufficient pressure to achieve arterial occlusion [19,20]. Previous studies reported CAT Gen 7 and SOFTT-W Gen 4 occlusion pressures of 175 mmHg and 104 mmHg, respectively [21]. A valuable reference for interpreting our results comes from recent laboratory studies of the Ukrainian DNIPRO Gen 2 and SICH tourniquets. Wall et al. demonstrated that the DNIPRO Gen 2 and the SICH reach complete arterial occlusion under controlled laboratory conditions, although at higher pressures compared with the CAT and the SOFTT W [22]. These findings are consistent with the high occlusion success rates found in our study for the DNIPRO Gen 2 and the SICH and may also explain the higher level of pain reported by our participants for the DNIPRO Gen 2.

Regarding speed of application, the median application times for the CAT Gen 7, the DNIPRO Gen 2, and the SICH in our study were similar to the application times reported for the CAT Gen 7 and the SOFTT by Potac et al., which were 31 s and 64 s, respectively [18]. Similarly to our study, a study by Treager [16] showed that the SOFTT-W was slower to apply than the other models tested.

The level of pain associated with tourniquet application emerged as an important parameter differentiating between the tourniquets tested. The DNIPRO Gen 2 was rated most painful, while the lowest pain levels were reported for the SICH and the SOFTT-W Gen 4. Pain during application has important clinical implications, particularly in self-aid scenarios under fire [21]. The higher level of pain associated with the application of the DNIPRO Gen 2 may be attributable to the design features of the tourniquet, including the lack of cushioning on the stabilising plate, the metal buckle with notches, and the rigid edges of the pressure plate, all of which may increase soft tissue compression beneath the tourniquet.

This study has a number of limitations that should be considered when interpreting its findings. First, all the tourniquets were used multiple times by the same participants, which does not reflect the single use nature of clinical tourniquets. Second, the participants’ prior experience with the tourniquet models tested was not assessed before the study. Instead, only a one-hour training session was provided, which may have influenced the results. Additionally, a formal sample size or power calculation was not performed prior to the study, as the number of participants was determined by the size of the available training group. It should also be noted that some of the calculated odds ratios were associated with relatively wide confidence intervals, which likely reflects the limited sample size and suggests that these estimates should be interpreted cautiously. Additionally, the order of tourniquet application was not randomised, which may have introduced a potential learning effect that could influence parameters such as application time or perceived ease of use. Kragh et al. note that familiarity with CAT application technique shortens the application time of the tourniquet and improves its effectiveness [23]. Further studies should include assessment of material quality and structural durability, which may be relevant for maintaining occlusion pressure over time, as well as evaluation of tourniquet performance when applied over tactical or winter clothing—factors that may significantly affect speed of application and user pain tolerance [24,25]. Moreover, the observed difference in occlusion success between CAT Gen 7 and DNIPRO Gen 2 in lower extremity application should be interpreted cautiously, as the study was conducted in a simulated experimental setting with repeated applications. In addition, the present study did not assess potential complications associated with tourniquet use, which should be addressed in future clinical studies conducted under real-world conditions.

## 5. Conclusions

The scale of injuries observed during the Russia–Ukraine conflict underscores the critical role of tourniquets as a primary tool for controlling massive extremity haemorrhage among both military personnel and civilian casualties. In the present study, the Ukrainian-manufactured DNIPRO Gen 2 and SICH tourniquets demonstrated occlusion success rates similar to those of the CAT Gen 7 and in several evaluated parameters outperformed the SOFTT-W Gen 4. The results suggest that domestically produced tourniquets may serve as a valuable alternative in situations where access to CoTCCC-recommended equipment is limited.

Our findings suggest that the DNIPRO Gen 2 and SICH tourniquets demonstrate comparable performance to CoTCCC-recommended tourniquets in achieving arterial occlusion in a controlled preclinical setting involving healthy volunteers under simulated conditions.

## Figures and Tables

**Table 1:** Comparison of arterial occlusion success and secondary outcomes between the tested tourniquet models.

Variable	Tourniquet Model			
	CAT Gen 7	DNIPRO Gen 2	SICH	SOFTT-W Gen 4
Arterial occlusion confirmed by Doppler—arm, *n* (%)	48 (94.1)	47 (94.0)	48 (98.0)	29 (61.7)
Arterial occlusion confirmed by Doppler—leg, *n* (%)	48 (94.1)	41 (80.4)	44 (86.3)	34 (68.0)
Application time (arm), s, Mdn (IQR)	32 (25–36)	36 (30–40)	32 (26–38)	35 (26–51)
Application time (leg), s, Mdn (IQR)	29 (24–34)	31 (24–34)	30 (24–38)	38 (30–45)
Pain intensity (arm), NRS, Mdn (IQR)	5 (3–6)	6 (4–7)	4 (3–6)	4 (3–6)
Pain intensity (leg), NRS, Mdn (IQR)	5 (3–6)	5 (4–7)	5 (3–6)	4 (4–6)
Windlass turns to occlusion (arm), Mdn (IQR)	2 (1–3)	2 (2–3)	3 (3–4)	3 (2–4)
Windlass turns to occlusion (leg), Mdn (IQR)	3 (2–4)	3 (3–4)	4 (3–4)	4 (3–5)
Ease of use (arm), 5-point Likert, Mdn (IQR)	4 (4–5)	4 (3–4)	3 (2–3)	2 (1–3)
Ease of use (leg), 5-point Likert, Mdn (IQR)	3 (3–4)	3 (2–3)	2 (2–3)	2 (1–2)

Mdn—median, IQR—interquartile range, NRS—numeric rating scale.

**Table 2:** Odds of successful arterial occlusion confirmed by Doppler ultrasound according to tourniquet model (mixed-effects logistic regression).

Tourniquet Model	Occlusion (by Doppler) Arm		*p*-Value	Occlusion (by Doppler) Leg		*p*-Value
	OR (Exp(B))	95% CI		OR (Exp(B))	95% CI	
SOFTT-W Gen 4	1 (reference)			1 (reference)		
DNIPRO Gen 2	9.66	2.60–36.85	<0.001	1.97	0.78–4.97	0.152
CAT Gen 7	9.85	2.66–36.55	<0.001	7.81	2.08–39.39	0.003
SICH	25.14	3.71–170.21	0.001	3.04	1.11–8.34	0.031

OR (Exp(B))—odds ratio calculated as the exponent of the logistic regression coefficient, 95% CI—95% confidence interval.

**Table 3:** Results of linear mixed-effect models comparing tourniquet models for application time, pain intensity, windlass turns to arterial occlusion, and ease of use.

Variable	Tourniquet Model	EMM	95% CI	Pairwise Comparisons (Bonferroni)
Application time—arm (s)	SOFTT-W Gen 4	41.0	19.8–62.1	SOFT > CAT (*p* = 0.034)
	DNIPRO Gen 2	37.3	16.2–58.5	
	CAT Gen 7	32.5	11.4–53.7	
	SICH	34.1	12.9–55.2	
Application time—leg (s)	SOFTT-W Gen 4	39.6	22.2–57.0	SOFT > DNIPRO (*p* = 0.007) SOFT > CAT (*p* < 0.001) SOFT > SICH (*p* = 0.007)
	DNIPRO Gen 2	31.6	14.2–49.0	
	CAT Gen 7	28.9	11.6–46.3	
	SICH	31.5	14.2–48.9	
Pain intensity—arm (NRS)	SOFTT-W Gen 4	4.6	1.7–7.5	ns
	DNIPRO Gen 2	5.7	2.8–8.5	
	CAT Gen 7	4.9	2.0–7.7	
	SICH	4.9	2.0–7.7	
Pain intensity—leg (NRS)	SOFTT-W Gen 4	4.5	1.6–7.5	DNIPRO > SOFT (*p* = 0.013) DNIPRO > SICH (*p* = 0.011)
	DNIPRO Gen 2	5.8	2.8–8.7	
	CAT Gen 7	5.0	2.1–8.0	
	SICH	4.5	1.6–7.4	
Windlass turns to occlusion—arm	SOFTT-W Gen 4	3.1	1.6–4.6	SOFT > DNIPRO (*p* = 0.015) SICH > DNIPRO (*p* < 0.001) SICH > CAT (*p* < 0.001) SOFT > CAT (*p* < 0.001)
	DNIPRO Gen 2	2.5	1.0–3.9	
	CAT Gen 7	2.3	0.8–3.7	
	SICH	3.3	1.9–4.8	
Windlass turns to occlusion—leg	SOFTT-W Gen 4	4.0	2.6–5.3	SOFT > CAT (*p* < 0.001) SICH > CAT (*p* < 0.001) SOFT > DNIPRO (*p* = 0.014)
	DNIPRO Gen 2	3.4	2.0–4.7	
	CAT Gen 7	3.1	1.9–4.5	
	SICH	3.8	2.5–5.2	
Ease of use—arm	SOFTT-W Gen 4	2.3	0.8–3.9	CAT > SICH (*p* < 0.001) CAT > SOFT (*p* < 0.001) DNIPRO > SICH (*p* < 0.001) DNIPRO > SOFT (*p* < 0.001)
	DNIPRO Gen 2	3.5	2.0–5.0	
	CAT Gen 7	4.1	2.6–5.5	
	SICH	2.6	1.1–4.1	
Ease of use—leg	SOFTT-W Gen 4	1.7	0.4–3.0	CAT > DNIPRO (*p* = 0.026) CAT > SICH (*p* < 0.001) CAT > SOFT (*p* < 0.001) DNIPRO > SICH (*p* = 0.004) DNIPRO > SOFT (*p* < 0.001)
	DNIPRO Gen 2	2.8	1.5–4.1	
	CAT Gen 7	3.3	2.0–4.6	
	SICH	2.2	0.9–3.5	

EMM—estimated marginal mean, CI—confidence interval, NRS—numeric rating scale, ns—not significant.

## Data Availability

The data presented in this study are available on request from the corresponding author.

## Abbreviations

ATO	Anti-Terrorist Operation
CAT	Combat Application Tourniquet
CI	Confidence Interval
COINs	Counter-Insurgency Operations
CoTCCC	Committee on Tactical Combat Casualty Care
CUF	Care Under Fire
GWOT	Global War on Terror
IQR	Interquartile Range
Mdn	Median
NATO	North Atlantic Treaty Organisation
NRS	Numerical Rating Scale
ORs	Odds Ratio
POI	Point of Injury
RMT-T	Ratcheting Medical Tourniquet–Tactical
SAM-XT	SAM Extremity Tourniquet
SOFTT-W	SOF Tactical Tourniquet–Wide
TMT	Tactical Mechanical Tourniquet
